# Efficient photocatalytic reactors *via* 3D printing: SLA fabrication and TiO_2_ hybrid materials†

**DOI:** 10.1039/d4ra07121b

**Published:** 2025-01-23

**Authors:** Isabel S. O. Barbosa, Yaidelin A. Manrique, Diana Paiva, Joaquim L. Faria, Ricardo J. Santos, Cláudia G. Silva

**Affiliations:** a LSRE-LCM – Laboratory of Separation and Reaction Engineering – Laboratory of Catalysis and Materials, Faculty of Engineering, University of Porto Rua Dr Roberto Frias 4200-465 Porto Portugal isob@fe.up.pt cgsilva@fe.up.pt; b LEPABE – Laboratory for Process Engineering, Environment, Biotechnology and Energy, Faculty of Engineering, University of Porto Rua Dr Roberto Frias 4200-465 Porto Portugal; c ALiCE – Associate Laboratory in Chemical Engineering, Faculty of Engineering, University of Porto Rua Dr Roberto Frias 4200-465 Porto Portugal

## Abstract

Additive Manufacturing (AM) was evaluated as a promising technology for constructing photocatalytic reactors due to its inherent ability to produce complex geometries with high precision and customization. In this work, a 3D structure was designed to achieve a good light distribution inside a cylindrical batch reactor and printed using the stereolithography (SLA) technique. A hybrid material composed of a commercial photoreactive resin (Formlabs Clear V4) and the benchmark photocatalyst TiO_2_ P25 Evonik (1 wt%) was prepared and characterized by scanning electron microscopy (SEM) and rheological and mechanical methods. To evaluate the photocatalytic activity of the materials, several experiments on the photocatalytic degradation of Rhodamine B (Rh_B_) were carried out using the 3D printed structure. Its performance was assessed by monitoring the concentration at specific times. Overall, the results demonstrate a simple, cost-effective, and fast technique to immobilize catalysts used in photocatalytic applications.

## Abstract 2

Additive Manufacturing using SLA was used to create 3D-printed photocatalytic reactors with TiO_2_-P25, achieving efficient RhB degradation. This approach offers a cost-effective, rapid method for immobilizing photocatalysts for several applications.

## Graphical abstract

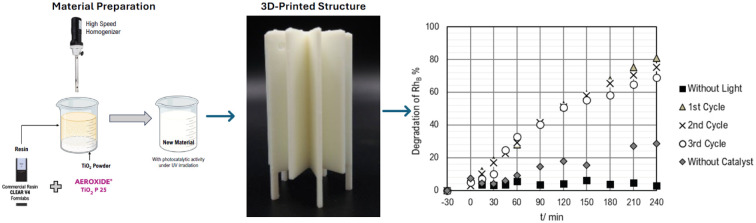

## 1 Introduction

Additive Manufacturing (AM) is a fast-prototyping technology that is useful in many engineering areas, namely chemical,^1–3^ environmental^4,5^ and biotechnological.^4^ The first report on this technology was in the 1980s when H. Kodama proposed a method for the quick automatic fabrication of a 3D plastic model using liquid photo-hardening polymer at a low cost and without excessive manual labour.^6^

Besides the low cost and simple operation, the 3D printing technology has other pros compared to the traditional methods since it can be used to quickly fabricate complex structures that are difficult, even impossible, to prepare using conventional methods.^7,8^ The design of the 3D object is more straightforward, more independent, and flexible since the structures are designed through computer-aided modelling; thus, it is easy to adapt the model design and printer parameters.^9–12^ Also, this technique enabled the development of a range and types of materials for several purposes.^13,14^

In the last few years, there has been a rapid development of 3D printing technology, improving the materials and processes to build 3D structures. In the chemical engineering field, this development enabled the manufacture of optimized reactor geometries in a short time with a great price/quality ratio. As well, over the last decade, the range of materials available for 3D printing has been studied, including ceramics, polymers (nylon, polypropylene, polyetheretherketone), metals (titanium, steel), and carbon-based materials.^9,15–17^ Some materials were also applied as catalyst supports^15,18,19^ and for reactors manufacturing.^1,15^ Most of the advances in 3D printing for chemical engineering are related to the process intensification concept, which seeks an efficient and sustainable route to produce chemical products.^15,20^ The main application of AM for process intensification lies in flow chemistry technology, *i.e.*, chemical reactions are performed in continuous flow processes within narrow channels.^21–23^ These chemical reactions can make use of heterogeneous catalysts and/or can be assisted by light, in the case of photocatalytic reactions.

In 2016, the application of AM in photocatalysis was successfully demonstrated by Castedo *et al.*,^1^ using a silicone microreactor with microchannels coated with an Au/TiO_2_ photocatalyst for hydrogen production. Since then, there has been more interest in the combination of these two technologies, specifically for water treatment and hydrogen production.

Hernández-Afonso *et al.* reported the production of 3D porous structures in CaSO_4_, which can be activated with TiO_2_ for photocatalytic degradation of pollutants in wastewater.^5^ Torres Arango *et al.* used sacrificial templating with direct foaming to synthesize multiscale porous TiO_2_ foams for photocatalytic processes.^24^ Lee *et al.* propose the fabrication of metal-based 3D printed photoelectrodes with conical arrays to perform photoelectrochemical water splitting enhanced by the direct growth of TiO_2_ nanotubes on this platform.^25^ In Jo *et al.* study, a hierarchical gyroid structure with embedded TiO_2_ nanoparticles was printed by fused deposition modelling using functionalized photodegradable polylactic acid (PLA) and its ability for photocatalytic applications was proved.^26^ Chen *et al.* developed a TiO_2_-based ink to manufacture porosity-tunable hierarchical 3D architectures with well-designed patterns for artificial photosynthesis *via* CO_2_ reduction.^27^ He *et al.* proposed a 3D printing method to manufacture g-C_3_N_4_ based hybrid aerogel membranes with patterned macroscopic architectures for solar wastewater remediation.^28^ Also, for photocatalytic water treatment, ZnO-based hierarchical structures on the 3D-backbone were proposed to be printed using the FDM (Fused Deposition Modelling) technique.^29^ Martín de Vidales *et al.* found that low-density-polyethylene (LDPE) can be a support for TiO_2_ in water remediation applications using the 3D printing FDM method since it presents lower density than water and high stability and resistance to degradation.^18^ For photogeneration of hydrogen, Elkoro *et al.* proposed 3D printed Au/TiO_2_ monoliths produced by additive superposition of microfilaments of titania-based pastes.^30^ TiO_2_-supported chitosan scaffolds (TiO_2_/CS) were proposed by Bergamonti *et al.* as a promising material for photocatalytic degradation of antibiotic pollutants in wastewater under UV-vis irradiation.^19^ Sevastaki *et al.* produced 3D photocatalytic structures made of nanocomposite polymeric filaments based on solid polystyrene enriched with TiO_2_ nanoparticles using an FDM 3D printer.^31^ For photooxygenation and photoredox catalysis, Hansen *et al.* reported a flow photochemical reactor manufactured by the VAT-based 3D printing of an isocyanate-functionalized acrylate monomer.^32^ Mai *et al.* presented a new 3D printing composite containing 10 wt% of modified TiO_2_ and 90 wt% of PLA for the degradation of organic compounds in wastewater by photocatalysis.^33^ The work of Do *et al.* introduced a composite of a styrene–acrylate (DC668) polymeric binder and the TiO_2_ brookite nanoparticles (NPs), which was immobilized on the surface of the glass substrate by a direct-ink-writing technique using a 3D printer for application in wastewater treatment.^34^

Recently, monoliths printed with stereolithography (SLA) technique using metal–organic frameworks (MOFs) have been under special attention due to the ability to enhance catalytic performance and to improve various aspects such as broadening the spectral response range and increasing porous and surface areas.^35,36^ The application of SLA-based 3D printing technology for fabricating photocatalytic reactors dates back to Vyatskikh *et al.* 2018 study.^37^ This research introduced the concept of polymer-derived titania, where a TiO_2_ precursor was combined with a photosensitive resin, 3D printed and then calcined to create a functional photocatalytic reactor. Another study by Huang *et al.* utilized 3D printing to create flexible supports with negative Poisson's ratios (NPR), enhancing both photocatalytic and mechanical properties. These supports were modified with TiO_2_, Ag nanowires, and metallization.^38^ Furthermore, the work on photocatalytic reactors *via* Digital Light Processing (DLP) of Chen's group demonstrated the use of VAT photopolymerization (VP)-based 3D printing to fabricate high-performance photocatalytic reactors using TiO_2_ nanoparticle slurries with enhanced photoefficiency.^39^

There are several AM techniques; however, this work is focused on stereolithography methodology. SLA belongs to a family of additive manufacturing known as VAT photopolymerization. The principle of this technique is the use of a light source (a laser or a projector) to cure liquid resin into plastic by a photopolymerization reaction to obtain the desired structures. When the laser excites the photoinitiator component, radical groups at the end of a molecule chain turn into free radicals that then join two polymer chains into one continuous group.^40^ Thus, a liquid tank filled with polymeric resin, which contains photo-initiators, is irradiated by a beam of light that goes across it, and a selective curing process occurs in the surface areas hit by the ray trajectory.^41^ The selective curing process is usually processed in a layer-by-layer manner in the platform plane and the laser draws each layer with high precision and accuracy.^40^ The piece is sustained by supports that promote the correct building of the structure and its attachment to the building bed. Once the printing process is finished, there are several steps to obtain the most consistent 3D printed structures. Cleaning in a tank with isopropyl alcohol to remove any uncured resin from its surface is the first step. After that, some materials require post-curing, a process which helps parts to reach their highest possible strength and stability. Finally, it is necessary to remove supports from the parts. Post-curing is particularly important for functional resins for engineering.

Several 3D printing methods show potential for use in photocatalytic technology, by directly printing active photocatalytic materials or creating photocatalytic substrates capable of immobilizing catalysts.^38,42–44^ One approach involves 3D-printing porous or tailored substrate structures, which are subsequently coated with photocatalytic materials.^43^ Another method is the direct 3D printing of composite materials that incorporate photocatalysts, such as TiO_2_, allowing the fabrication of multifunctional devices with complex geometries.^37^ Finally, pure photocatalytic materials can be used to create 3D-printed structures entirely composed of active catalysts, maximizing surface area and avoiding catalyst detachment issues. Advances in materials, such as photocurable resins infused with photocatalytic materials, and printing techniques, have significantly expanded the potential for designing efficient, application-specific photocatalytic systems.

For direct printing of active photocatalytic materials, 3D printing technology is used to fabricate structures made of these materials. These materials usually have properties that allow them to utilize light energy for catalytic reactions. Techniques like stereolithography can deposit photocatalytic materials layer by layer with precision, creating detailed structures tailored for particular purposes. This approach provides precise control over material composition and structure, enabling the design of customized photocatalytic systems that are optimized for efficiency and performance. SLA technology combined with integral printing photocatalysts has found applications in the fields of environment and energy.^45^ The preferred 3D printing technology is SLA, particularly for fabricating continuous flow reactors or when pressure drop is a critical parameter. SLA offers advantages over other additive manufacturing (AM) methods, as the individual layers are covalently crosslinked, ensuring isotropy in all three dimensions without macroscopic grain structures or voids.^46^ Consequently, SLA-printed structures exhibit sufficient mechanical robustness.^47^

The integral printing process is relatively straightforward and widely applicable. However, it's crucial to carefully control the ratio of photocatalyst to precursor to maintain the desired rheological properties of the material during printing.^42^ Not all 3D printing methods can seamlessly incorporate both substrate and catalyst into active materials. Sometimes, the active components may get covered by the polymer, impacting the efficiency of catalysis and adsorption.

In summary, 3D printing technology offers a versatile platform for advancing photocatalytic technology. It allows for the precise fabrication of structures customized to meet the specific needs of photocatalytic reactions.

However, current technological gaps in the field of photocatalytic reactor design include the difficulty in achieving complex geometries that optimize light distribution and maximize photocatalytic efficiency. Traditional manufacturing methods often struggle to create intricate structures that enhance the interaction between light and photocatalysts, limiting their effectiveness in real-world applications. Additionally, reactor designs that improve photocatalytic activity through optimized geometries are often challenging to implement without compromising material stability or scalability. These limitations hinder the development of highly efficient, customizable photocatalytic reactors. In this work, these challenges are addressed by utilizing stereolithography (SLA) 3D printing to create reactors with complex, optimized geometries that require no additional chemical post-treatment, providing an efficient and cost-effective solution for enhanced photocatalytic performance. A commercial resin, Clear V4 (Formlabs), was impregnated with titanium dioxide (TiO_2_) to manufacture 3D structures with photocatalytic activity that can be applied in photocatalytic reactions for several applications. These structures were designed to allow light to reach the system and were printed using the SLA methodology. They can be considered stable and reliable supports for catalysts. The photocatalytic activity of the materials was experimentally benchmarked using the degradation of Rhodamine B (Rh_B_).

## 2 Materials and methods

### 2.1 Materials

The transparent commercial resin, Clear V4 (Formlabs), was used to print 3D structures with photocatalytic activity under ultraviolet irradiation. Titanium dioxide (TiO_2_) Aeroxide® P25 (Evonix) was selected as the catalyst due to its well-known good performance in photocatalytic reactions for several applications. This material is a fine-particulate powder, which contains a combination of anatase (*c.a.* 80%) and rutile crystal structure and presents a surface area of 50 m^2^ g^−1^, high purity (≥99.50%), and good thermal and chemical stability. The physical and chemical properties of TiO_2_ Aeroxide® P25 (Evonix) are described in ESI.†

Clear V4 resin is a photoreactive liquid mixture of methacrylic acid esters and a photoinitiator produced by Formlabs, Inc.®. More specifically, this mixture is composed of methacrylated oligomer, methacrylated monomer and diphenyl (2,4,6-trimethylbenzoyl) phosphine oxide (<1%). This resin is chemically stable, doesn't undergo hazardous reactions, and does not produce hazardous decomposition products under normal conditions of storage and use. Information on the physical and chemical properties of the resin is summarized in Table 1.

**Table 1:** Physical and chemical properties of Clear V4 resin (manufacturer data)

Physical state	Liquid
Colour	Yellow
Odour	Light/characteristic/acrylate
Initial boiling point and boiling range	>100 °C
Flash point	Closed cup: >93.333 °C
Relative density	1.09 to 1.12
Solubility(ies)	Very slightly soluble in the following materials: cold water and hot water
	Soluble in organic solvents
Viscosity	Dynamic (room temperature): 850 to 900 mPa s

### 2.2 Dispersion of TiO_2_ into the commercial resin

**Fig. 1:**
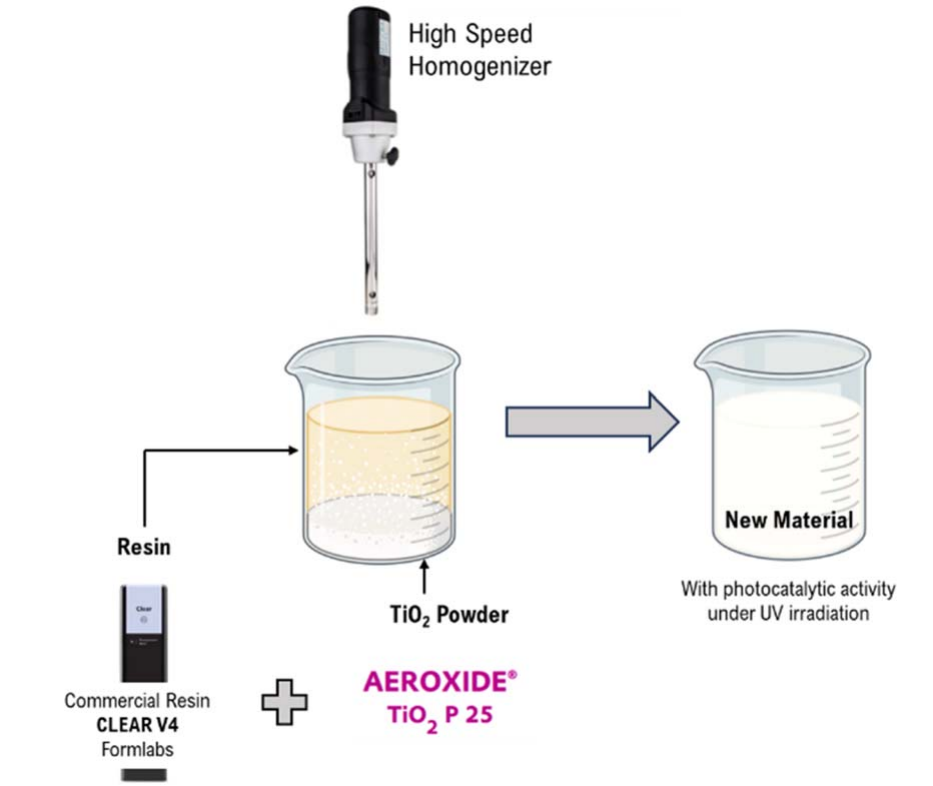
Dispersion process of TiO_2_ powder on Clear V4 commercial resin.

For the production of the hybrid material composed of Clear V4 resin and TiO_2_, the latter was dispersed in 130 g of the resin using a high-speed homogenizer, minibatch D-15 (Miccra), for 10 minutes at 39 000 rpm. Two materials were prepared by varying the load of TiO_2_ in the dispersion (0.1 and 1.0 wt%). A schematic representation of the dispersion process is illustrated in Fig. 1.

The rheological properties of both dispersions were assessed and compared to the properties of the starting resin. Rheological tests were performed on Anton Paar® Rheometer MCR 92 SN82478971 operating at continuous rotation using a plate/plate geometry measuring system (diameter – 50 mm, zero gap – 0.500 mm). The flow behaviour was assessed by controlled shear rate (CSR) and controlled shear stress (CSS) tests. Also, for the evaluation of temperature–dependent flow behaviour, a rotational test was carried out under constant shear conditions. For temperature control, the temperature–dependent flow behaviour was performed with a Peltier element, which can be coupled to the rheometer. The characteristics of the rheological tests are described in ESI.†

### 2.3 3D-printing and characterization of the photocatalytic structures

A stereolithography (SLA) printer, Form2 (from Formlabs), was used to manufacture all the structures addressed in this work. The quality of the printing was defined by several parameters, namely the properties of the 3D printer, which are described in Table 2.

**Table 2:** Properties of SLA 3D-printer Form2 (manufacturer data)

Technology	Stereolithography (SLA)
Printer	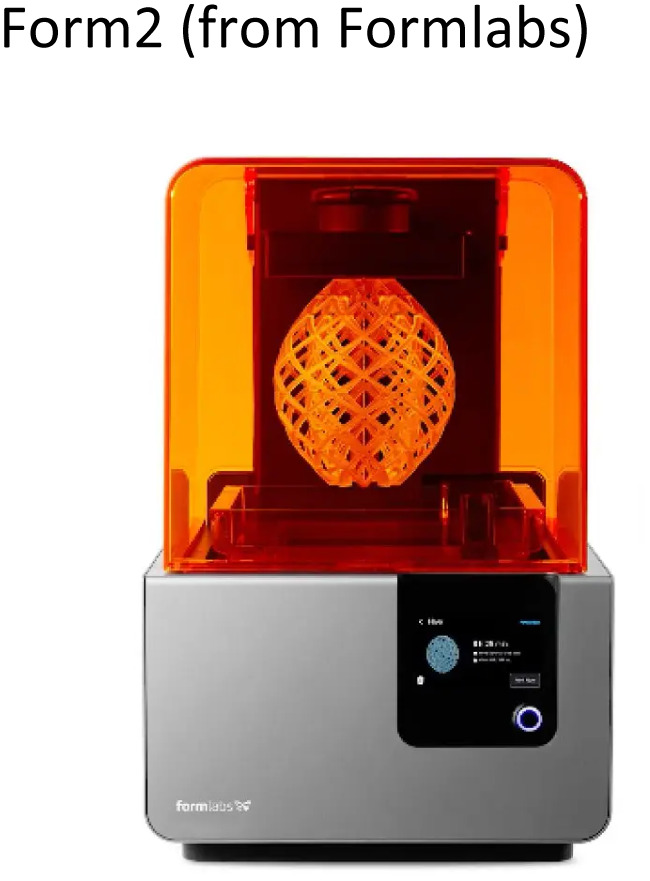
Peel mechanism	Sliding peel process with wiper
Build volume	145 × 145 × 175 mm
Layer thickness (axis resolution)	25, 50, 100 microns
Laser spot size (FWHM)	140 microns
Laser specifications	EN 60825-1:2007 certified Class 1 laser product 405 nm violet laser 250 mW laser
Operating temperature	Auto-heats to 35 °C
Supports	Auto-generated
	Easily removable

The prototypes were designed using Solidworks® software, and the respective STL file was imported to the print preparation software, PreForm®. The layer thickness was fixed at 50 μm, and the supports were auto-generated. First, samples were printed at angles of 0°, 45° and 90° using resin dispersions containing 0.1% or 1% of TiO_2_. In an SLA 3D printer, printing angles refer to the orientation of the layers relative to the build platform. At 0°, the layers are parallel to the platform, resulting in a smooth finish but often needing more support structures. At 45°, the layers are angled, which balances support needs, improves layer adhesion, and reduces layer line visibility. At 90°, the layers are perpendicular, potentially reducing support for vertical features but increasing the layering effect and affecting mechanical properties. By changing the printing angles, it is possible to evaluate the impact of orientation on the quality, mechanical properties, and efficiency of materials containing TiO_2_.

The parts were printed using the “Open Mode” option, which allows for the use of non-standard resins not recognized by the printer. Usually, the resin fill system is automated, but since the dispersion used was not a standard material, the automatic fill system had to be turned off. This means the resin had to be manually filled and monitored during the printing process, ensuring that the printer could work with the custom resin dispersion. Once the printing was completed, parts were cleaned using isopropyl alcohol and then cured using a Form Cure device (from Formlabs) emitting at 405 nm, under a temperature of 60 °C for 1 h.

The morphology of the samples obtained at different printing angles and containing different TiO_2_ loads was characterized by optical microscopy, using a Zeiss microscope coupled with an Axiocam 105 colour camera. The micrographs were obtained at resolutions of 50× and 100×. Higher magnification micrographs were obtained by scanning electron microscopy coupled with EDS (Phenom ProX). Furthermore, FTIR (Fourier-transform infrared spectroscopy) and UV-vis spectroscopic analysis of printed parts were carried out using a JASCO V-680 FTIR spectrometer equipped with a DLATGS detector and JASCO V-560 UV-vis spectrometer, respectively.

Dynamic mechanical analysis (DMA) was performed using a DMA 242 E Artemis (NETZSCH-Geratebau GmbH). The dimensions of the samples used for these assays were 60 × 8 × 2 mm. Measurements carried out in duplicate were performed in 3-point bending mode with a frequency of 1 Hz at temperatures ranging from 0 to 100 °C.

The samples for optical microscopy, SEM/EDS (10 × 8 × 2 mm) and DMA assays (60 × 8 × 2 mm) were printed in triplicate for the three-orientation angles of 0°, 45° and 90°.

### 2.4 Photocatalytic tests

**Fig. 2:**
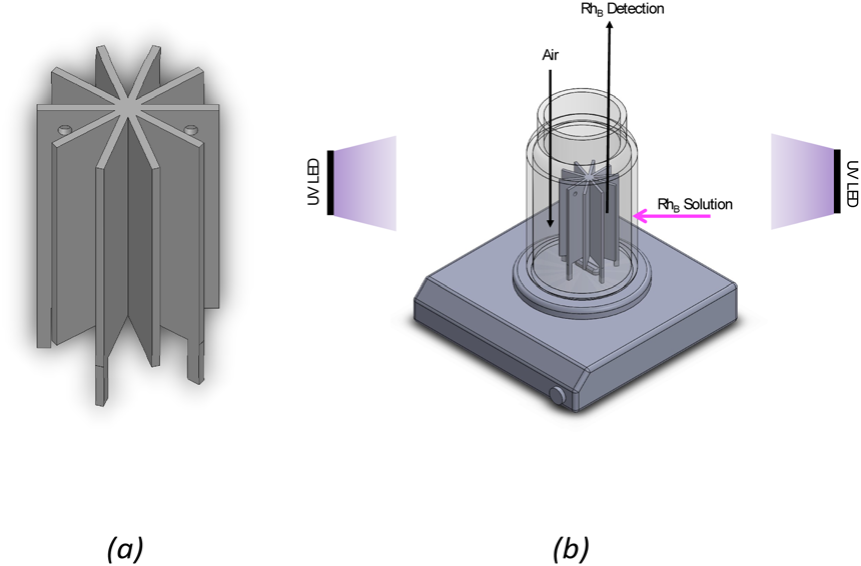
(a) Design of 3D catalytic support of SolidWorks and (b) schematic representation of experimental setup of photocatalytic degradation of Rh_B_.

The photocatalytic activity was assessed for the photocatalytic degradation of Rhodamine B (Rh_B_) under UV irradiation. The experiments were carried out using a star-shaped 3D printed structure produced at 90° and containing 1.0 wt% TiO_2_ (Fig. 2a). The 3D structure was designed to achieve the best light distribution inside a cylindrical batch. In a typical experiment, the structure was placed inside a cylindrical glass reactor filled with 70 mL of a 10 μM Rh_B_ aqueous solution, which was continuously stirred and purged with air. Fig. 2b shows a scheme of the experimental setup. The system was left under dark conditions for 30 min to establish an adsorption–desorption equilibrium between Rh_B_ and the photocatalyst. Then, a 4-UV LED system placed around the reactor was turned on (*λ*_max_ = 370 nm; irradiance ≈ 130 W m^−2^ each LED), and the photocatalytic degradation of Rh_B_ was followed by analysing the UV-vis spectrum of the solution over time using a JASCO V-560 spectrometer. The LED intensity was measured at 3 cm from the reactor walls using a UV-vis spectroradiometer device (USB2000+, Ocean Optics, USA).

### 2.5 Economic analysis

3D printing technology, used for designing and prototyping the photoactive structure employed in this work, incurs costs that differ from those associated with traditional manufacturing methods. Here, a comparison with traditional plastic manufacturing techniques, such as RIM (Reaction Injection Molding) and thermoplastic injection moulding, was carried out. RIM involves the mixing of two or more monomers, typically a polyol and an isocyanate, which react through polymerization and expand in a mould to form a plastic part.^48^ This process is ideal for creating large, complex parts with varying wall thicknesses, offering excellent surface finishes and lower tooling costs. Thermoplastic injection moulding (TIM), on the other hand, is a continuous process where thermoplastic materials (*e.g.* plastic pellets) are melted and forced through a die to create long parts with a consistent cross-sectional profile.^49^ This method is best suited for producing uniform profiles at high volumes. Therefore, using the typical costs of materials, design and engineering, and the mould for thermoplastic injection moulding and RIM techniques, an estimation of function costs was carried out.

## 3 Results and discussion

### 3.1 Rheological tests of the resin and dispersions

**Fig. 3:**
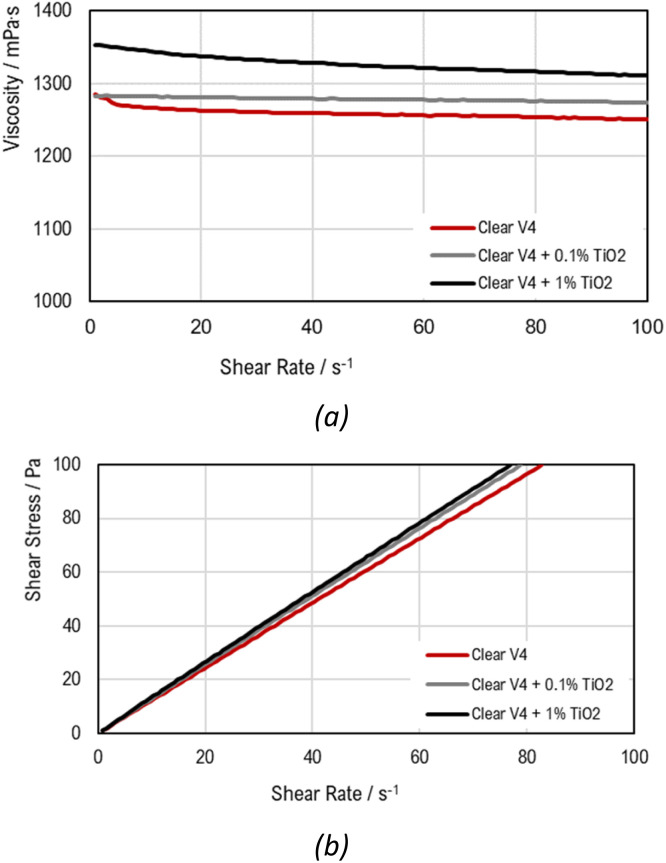
(a) Viscosity curve (CRS) and (b) flow curve (CSS) for Clear V4 resin and the dispersions.

The flow behaviour of the liquid Clear V4 resin and the dispersions was characterized by rotational tests. This behaviour is presented in the viscosity curves (Fig. 3a) (viscosity in function of shear rate or shear stress) and flow curves (Fig. 3b) (shear stress in function of shear rate).

**Fig. 4:**
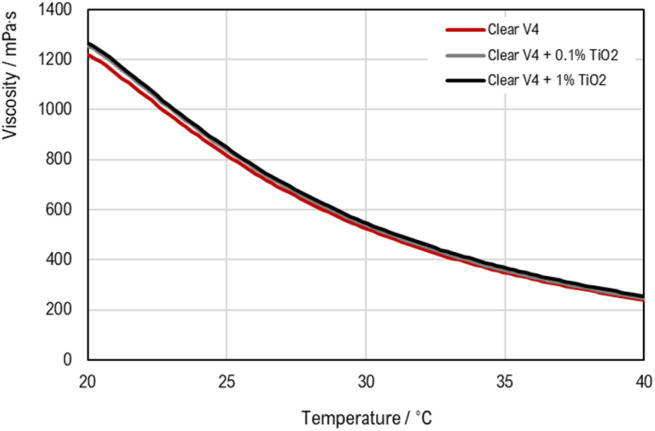
Temperature–dependent viscosity curves.

Also, the temperature–dependent flow behaviour is assessed from temperature–dependent viscosity curves with the viscosity as a function of temperature (Fig. 4).

The rheological results in Fig. 3a indicate that all the materials under investigation present Newtonian fluid behaviour, meaning their viscosity remains constant regardless of the shear rate. When TiO_2_ is added to the commercial resin, a slight increase in viscosity is observed. A percentual variation in viscosity of *c.a.* 5% was obtained between the commercial resin and the Clear V4 + 1% TiO_2_. This indicates that TiO_2_ makes the resin slightly thicker, though the change is relatively minor. Despite the addition of catalyst particles, the materials do not show yield stress, as illustrated in Fig. 3b. Yield stress is the minimum amount of stress required to initiate permanent deformation in a material. The lack of significant yield stress in the materials with catalyst particles suggests that these particles do not change the point at which the material starts to flow or deform under stress. This stability in flow characteristics is beneficial for ensuring consistent performance in SLA 3D printing. The main results are summarized in Table 3.

**Table 3:** Rheological results of rotational tests

	Clear V4	Clear V4 + 0.1% TiO_2_	Clear V4 + 1% TiO_2_
Viscosity VC (Pa s)	1.26	1.28	1.33
Viscosity FC (Pa s)	1.21	1.27	1.30
Yield stress (Pa)	0	0	0

Furthermore, analysis of temperature–dependent viscosity curves (Fig. 4) reveals that viscosity decreases with temperature for all three materials.

In 3D printing using SLA, the rheological properties of the resin are crucial for print quality. Proper viscosity is important for ensuring good wetting, meaning the resin adheres well to surfaces and coats them evenly. If the resin is too thick or too thin, it can lead to problems such as poor adhesion or excessive spreading.^50^ When titanium dioxide is added to the resin, the increase in the viscosity is not significant, which can ensure the resin's behaviour during printing. Additionally, the viscosity affects how bubbles in the resin escape. Higher viscosity can trap bubbles, leading to defects, while a well-balanced viscosity helps minimize bubble entrapment.^51^ The settling time of TiO_2_ particles is also significant. Longer settling times help maintain a stable resin mix by preventing particles from quickly settling out, which ensures consistent print quality.^52^ Stokes' Law, which describes how particles settle in a fluid, is relevant here. It shows that particles settle more slowly in higher-viscosity fluids, which is advantageous for keeping TiO_2_ particles well-dispersed in the resin. By optimizing these factors, including controlling the viscosity and ensuring good particle dispersion, the SLA 3D printing process can achieve high-quality, defect-free prints.

The rheological results showed that the incorporation of TiO_2_ into the commercial resin does not drastically alter its flow characteristics, maintaining its suitability for 3D printing applications. The similar flow behaviour observed in Clear V4, Clear V4 + 0.1% TiO_2_, and Clear V4 + 1% TiO_2_ indicate that they can all be effectively used in the 3D printing process without significant differences in printability or processing requirements. This consistency in flow behaviour simplifies the printing process and ensures reproducibility in the fabrication of photoreactors or other printed structures.

### 3.2 Morphology of 3D printed structures

**Fig. 5:**
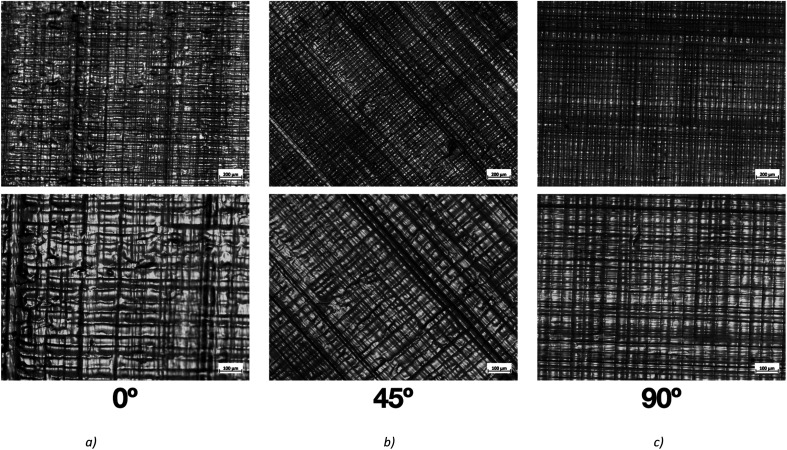
Optical microscopy images of the printed plane plate in Form2 from Formlabs. For the three angles: (a) 0° horizontal, (b) 45° and (c) 90° vertical. At a resolution of 50× and 100×, top images and bottom images, respectively.

**Fig. 6:**
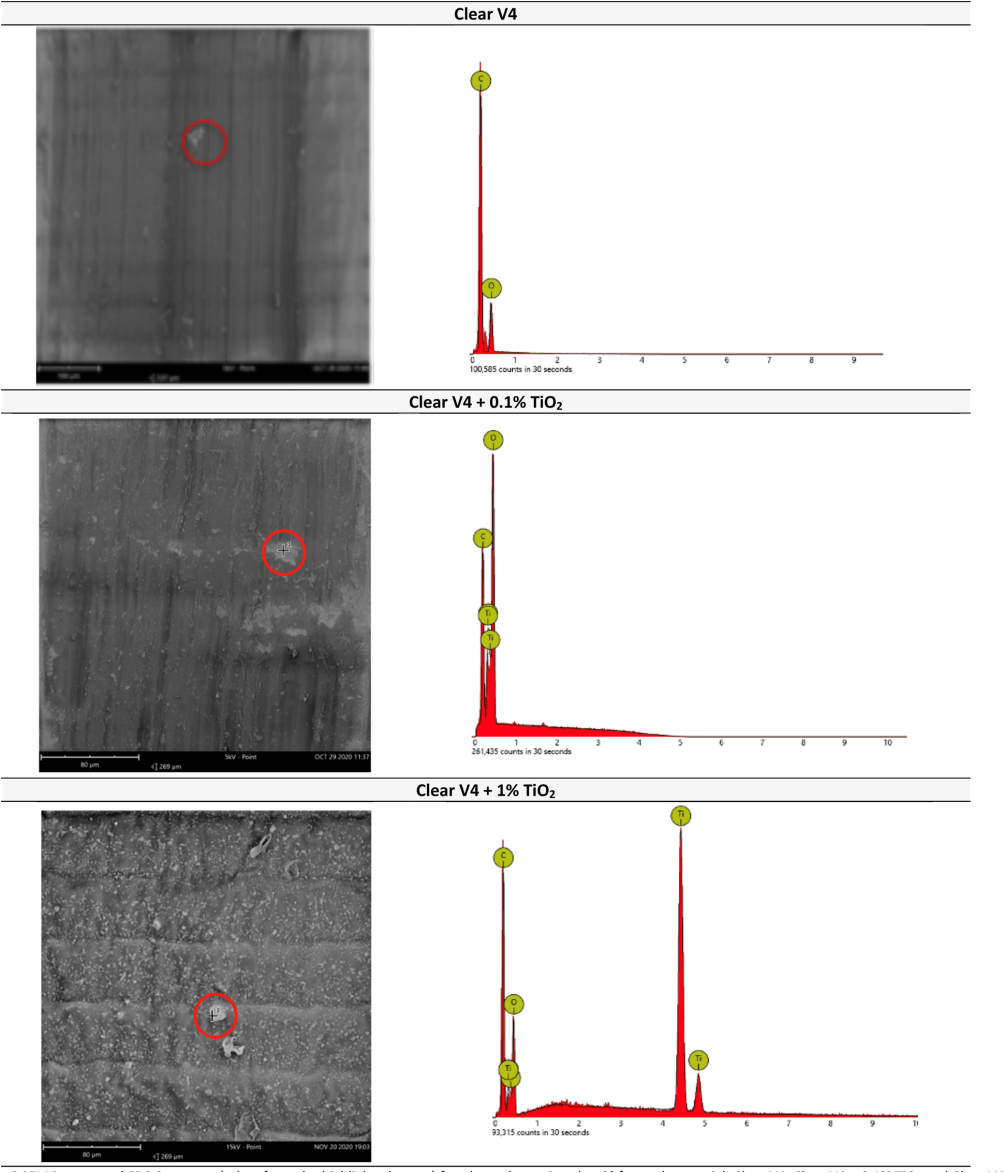
SEM images and EDS spectrum (taken from the highlighted zones) for plane plate printed at 0° for each material: Clear V4, Clear V4 + 0.1% TiO_2_ and Clear V4 +1% TiO_2_.

The morphology of the 3D printed parts was assessed from optical microscopy (Fig. 5) and SEM and EDS analysis (Fig. 6).

The observation of optical microscopy images proved that the printing layers maintain the orientation defined in the printer software and highlights the precision and accuracy of the 3D printing process. This indicates that the printer software effectively translates the digital design into physical layers during the printing process, ensuring that the desired geometry and structure are faithfully reproduced. The orientation of layers in 3D printing plays a crucial role in determining the mechanical properties, surface finish, and overall quality of the printed object. Proper layer orientation can enhance the strength and durability of the printed part, minimize the occurrence of defects such as warping or delamination, and optimize the printing time and material usage. In terms of material application, the orientation of layers can also influence the performance and functionality of the printed object.

The structural surface properties of the printed parts were assessed by SEM and EDS analysis. It was revealed the presence of titanium on the surface of structures printed with Clear V4 + 0.1% TiO_2_ and Clear V4 + 1% TiO_2_. However, the distribution of TiO_2_ was found to be heterogeneous, indicating uneven dispersion of the catalyst particles within the printed material. As expected, the structure printed with 1% TiO_2_ exhibited a higher concentration of titanium on the surface compared to the structure printed with 0.1% TiO_2_. This observation shows that the higher concentration of TiO_2_ leads to greater deposition of the photocatalyst at the surface of the printed part. Thus, this structure was selected for testing the photocatalytic activity. This decision is likely based on the expectation that the increased presence of TiO_2_ on the surface would enhance the photocatalytic performance of the printed part.

**Fig. 7:**
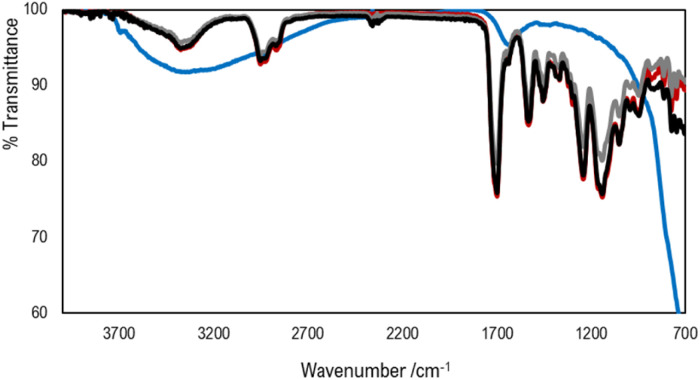
FTIR spectra of powder TiO_2_ (blue) and printed materials Clear V4 (red), Clear V4 + 0.1% TiO_2_ (grey) and Clear V4 + 1% TiO_2_ (black).

**Fig. 8:**
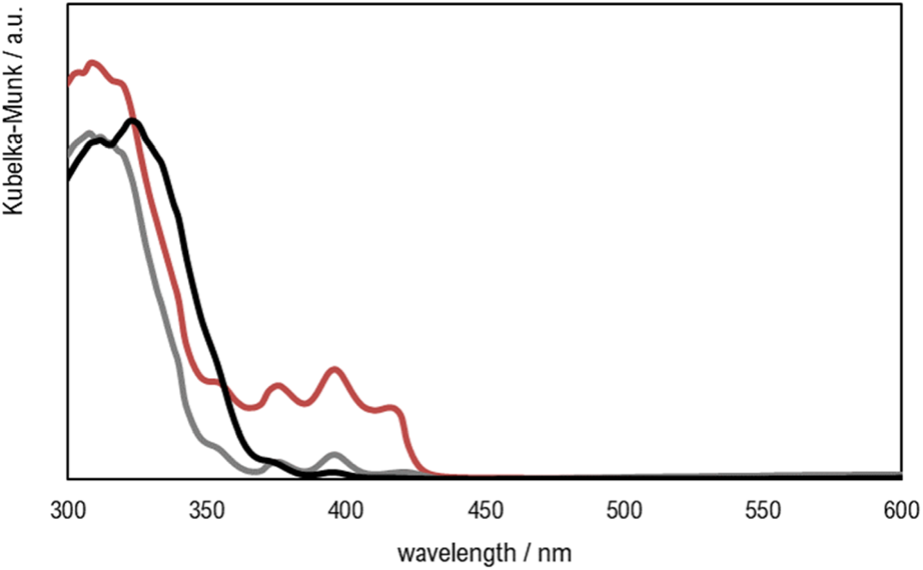
UV-visible spectra of printed materials Clear V4 (red), Clear V4 + 0.1% TiO_2_ (grey) and Clear V4 + 1% TiO_2_ (black).

FTIR-ATR and UV-vis spectra of TiO_2_ powder and the printed parts containing different photocatalyst loads are shown in Fig. 7 and 8, respectively.

The addition of TiO_2_ did not change the material transmittance spectrum of the base Clear V4, as is shown in Fig. 7, which may be attributed to the low amount of photocatalyst present at the surface of the hybrid material.

The UV-vis spectra of the resin and hybrid materials show absorbance in the typical wavelength regions of methacrylate and methacrylate polymers (<430 nm) and of TiO_2_ (<380 nm).^53^

It is important to highlight that the materials' absorbance and the SLA printer laser emission wavelength have to match to guarantee the success of the printing processes, especially when incorporating additives like TiO_2_. While the increased absorbance may pose challenges for printing parts with higher TiO_2_ content, the ability to maintain mechanical properties indicates the feasibility of producing functional printed parts with desired characteristics, even in the presence of such additives.

### 3.3 DMA tests – mechanical properties

**Fig. 9:**
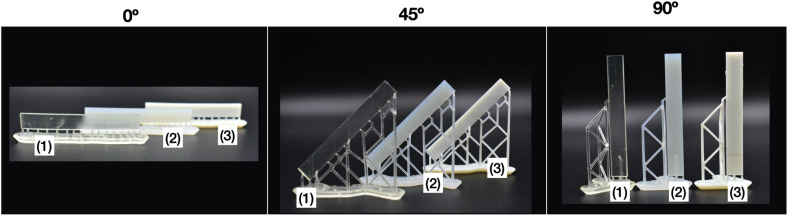
Photographs for materials printed at different printing angles, these plane plates were used for the DMA test. Legend: (1) Clear V4, (2) Clear V4 + 0.1% TiO_2_ and (3) Clear V4 + 1% TiO_2_.

Mechanical Properties were measured by Dynamic Mechanical Analysis (DMA). Fig. 9 shows the photographs of the samples prepared for DMA assays (60 × 8 × 2 mm), printed in triplicate for the three orientation angles of 0°, 45° and 90°, for each material.

From Fig. 9, it is easily observed that the presence of TiO_2_ in the resin promotes an opacity in the material. The addition of TiO_2_ to the resin introduces optical effects that reduce the transparency of the material, resulting in increased opacity.

**Fig. 10:**
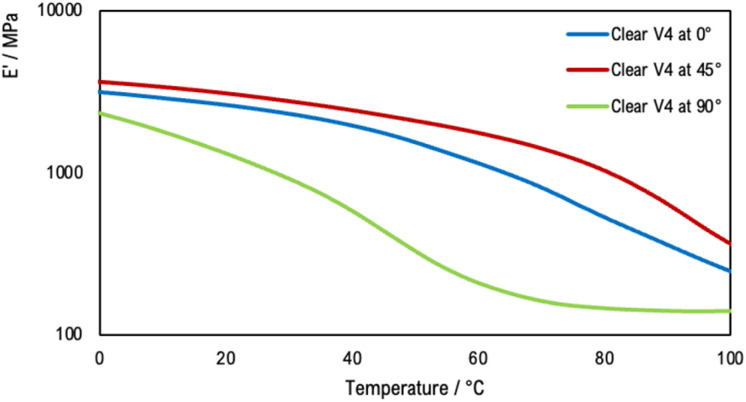
Tensile DMA thermograms for commercial resin Clear V4 (Formlab) printed at 0°, 45° and 90°.

**Fig. 11:**
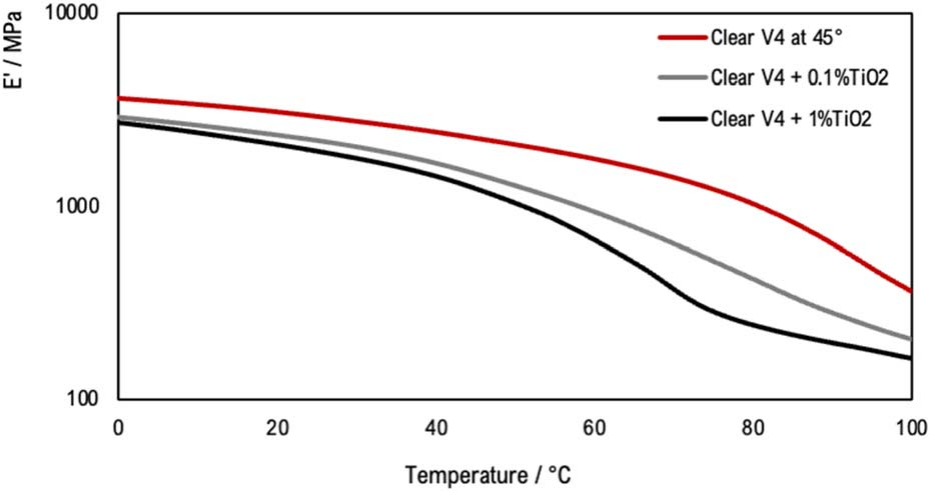
Tensile DMA thermograms for Clear V4, Clear V4 + 0.1% TiO_2_ and Clear V4 + 1% TiO_2_ printed at 45°.

Tensile DMA thermograms were plotted to study the mechanical behaviour of the 3D-printed samples. Fig. 10 compares the mechanical properties of the samples printed with the Clear V4 commercial resin for three orientation angles of 0°, 45° and 90°. Fig. 11 presents the tensile DMA thermograms for Clear V4, Clear V4 + 0.1% TiO_2_ and Clear V4 + 1% TiO_2_ printed at 45°.

The observed decrease in storage modulus and glass transition temperature in the resin containing 0.1% and 1% TiO_2_ suggests that the resin has limited affinity or interaction with the TiO_2_ particles. The decrease in storage modulus indicates a reduction in the material's stiffness and ability to resist deformation when modified with TiO_2_. Similarly, the decrease in glass transition temperature suggests a decrease in the temperature at which the material changes from a rigid to a more flexible state. These changes imply that the presence of TiO_2_ particles disrupts the polymer matrix, leading to a decrease in the material's mechanical properties and thermal stability. Despite these changes, the mechanical behaviour of the resin modified with TiO_2_ remains similar to that of the pristine resin. This suggests that while the addition of TiO_2_ affects certain mechanical properties, such as stiffness and thermal stability, it does not significantly alter the overall mechanical behaviour of the material.

### 3.4 Photocatalytic tests

**Fig. 12:**
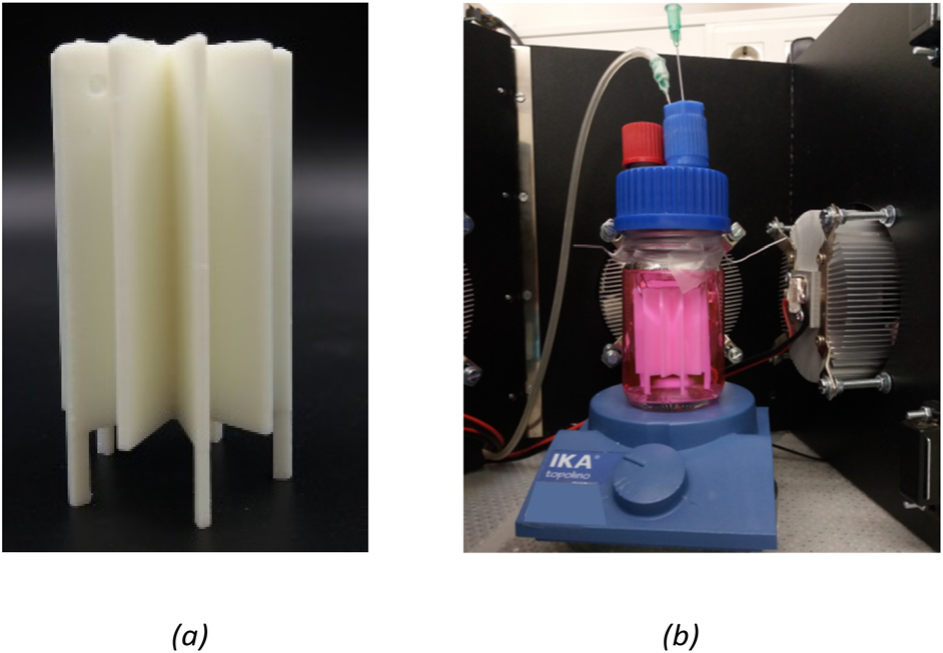
(a) 3D printed structure and (b) experimental setup of photocatalytic degradation of Rh_B_. (*T* = 20 °C; [Rh_B_]_in_ = 10 μM; *λ*_max_ = 370 nm; total irradiance ≈ 520 W m^−2^).

The photocatalytic experiments were carried out to assess the photocatalytic activity of the 3D-printed structure containing 1% of TiO_2_. TiO_2_ under UV irradiation and its photo-stability over multiple cycles of utilization. The 3D-printed star structure (Fig. 12a) was placed in the middle of a cylindrical glass reactor for photocatalytic tests and irradiated from the outside using a UV-LED system. Fig. 12b shows the experimental setup used to perform the photocatalytic degradation of Rh_B_ experiments.

**Fig. 13:**
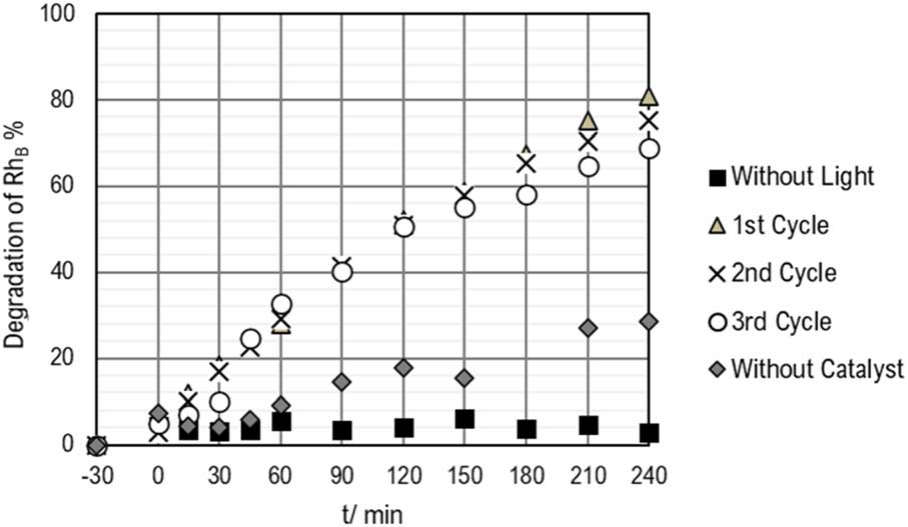
Photocatalytic degradation of Rh_B_ over 3 consecutive cycles.

The results of the photocatalytic degradation of Rhodamine B over time are summarized in Fig. 13, offering insights into the photocatalytic activity of the 3D-printed structure impregnated with TiO_2_ and its photo-stability across several utilization cycles.

In the absence of light, no degradation of Rh_B_ was observed, indicating that the reaction does not proceed without light activation. However, when the reactor was irradiated with 370 nm LEDs, corresponding to the activation wavelength for TiO_2_ photocatalysts, a significant 80% decrease in RhB concentration was observed after 240 minutes. This reduction in Rh_B_ concentration demonstrates the effectiveness of the TiO_2_ photocatalyst in degrading organic pollutants under UV irradiation. The slight increase in Rh_B_ degradation percentage observed in the 3D printed structure without the catalyst can be attributed to surface adsorption of Rh_B_ onto the structure. While this adsorption may contribute to some degree of degradation, it is much smaller than the photocatalytic degradation by TiO_2_, and after saturation, it will stop.

The photo-stability of the 3D-printed structure refers to its ability to maintain consistent photocatalytic activity over repeated cycles of utilization. The confirmation that the structure remains photo-stable for at least three utilization cycles indicates its reliability and durability for long-term applications. This suggests that the photocatalytic properties of the TiO_2_-containing 3D-printed reactor are robust and can be consistently relied upon for repeated use.

Overall, the results underscore TiO_2_-containing 3D-printed reactor for photocatalytic applications, as evidenced by the significant degradation of Rh_B_ under UV irradiation. Additionally, the confirmation of photo-stability over multiple utilization cycles is indicative of the applicability of the reactor for continuous and long-term use in photocatalytic processes.

### 3.5 Economic analysis

The typical costs used to determine the cost function to produce the 3D structure, for the three plastic manufacturing techniques (3D Printing, RIM and TIM), are detailed in Table 4.

**Table 4:** Typical costs of plastic manufacturing techniques

**3D printing**	
Cost of design and engineering	60€
Material cost (photopolymer resins)	20€ per kg

**RIM**	
Cost of the mold and engineering	5000€
Material cost (polyol and an isocyanate)	10€ per kg
Cycle time per 10 parts	5 min
RIM machine cost	20€ per h

**TIM**	
Cost of the mold and engineering	50 000€
Material cost (polyol and an isocyanate)	2€ per kg
Cycle time per 10 parts	1 min
TIM machine cost	20€ per h

From the data available in 3D printer software (PreForm®), the volume of resin necessary to build a structure is around 10 mL. Also, in 3D printer, a minimum amount of resin is needed in the tray (150 mL). The cost of the 3D printer or the 3D printer per hour was not considered because is negligible when compared with other techniques.

The cost functions were determined based on the costs presented in Table 4 and described for 3D printing in the following equation.
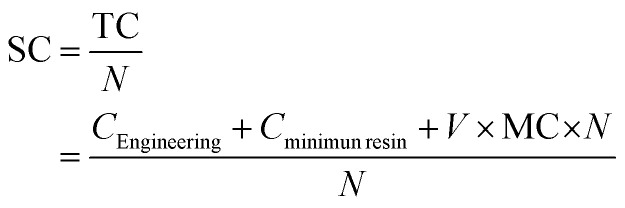
where SC is the specific cost of each part, defined as the total cost (TC) per number of parts (*N*). The total cost for 3D printing considers the cost of design and engineering (*C*_Engineering_), the cost of the minimum amount of resin in the tray for the printing process (*C*_minimum resin_), and the cost of the material cost per part, which depends on *V* is the volume of the part and MC is the material cost per kg.

For RIM and TIM, the cost function is in the following equation:
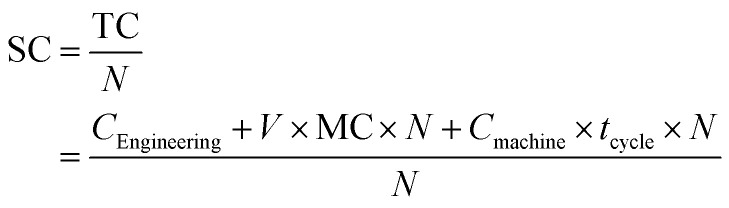
where *C*_machine_ is the cost of the manufacturing machine per hour and *t*_cycle_ is the time to produce a part.

**Fig. 14:**
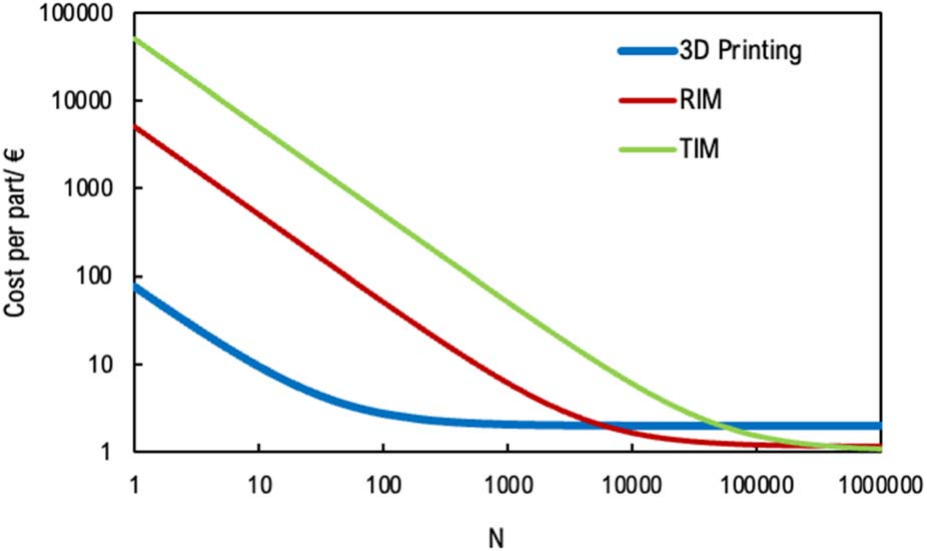
Comparison of costs per part for plastic manufacturing techniques (3D printing, RIM and TIM) on a logarithmic scale.

Fig. 14 presents the comparison of costs per part for each plastic manufacturing technique described above.

From Fig. 14, it can be concluded that 3D printing is the most viable technique for producing a relatively low number of parts, approximately 7000 units. This is due to its lower initial setup costs and flexibility in design modifications. However, as the production volume increases, the cost-effectiveness of 3D printing diminishes because its cost per part remains fixed at 2€. For larger production volumes, other manufacturing techniques, such as RIM or TIM, should be considered. These methods become more cost-efficient as the number of parts increases due to their lower variable costs. Specifically, for production runs exceeding 7000 parts, the price per part using RIM or TIM drops below the fixed cost of 3D printing. Moreover, when comparing RIM and TIM, RIM offers greater financial advantages for medium-scale production, particularly when the number of parts is below 500 000. In this case, 10 parts are injected per mould. Therefore, production volumes range from 1000 to 500 000 parts. Conversely, for production volumes exceeding 500 000 parts, TIM becomes more cost-effective due to its higher throughput and efficiency in handling large-scale manufacturing.

However, the characteristics of 3D-printed reactors, such as higher catalytic surface area per unit volume, optimized flow fields, and the ability to fabricate reactors with non-conventional geometries, are important when compared to other manufacturing tools and play a crucial role in the economic evaluation.

## 4 Conclusions

The capability of Additive Manufacturing (AM) to construct photoreactor prototypes was showcased by directly printing a material consisting of a commercial resin and a standard catalyst (TiO_2_) using an SLA 3D printer. The rheological characteristics of the photocatalytic-active resin indicated its suitability for 3D printing purposes. Through mechanical studies, it was established that the functional resin exhibits identical behaviour to the commercial one; thus, the addition of TiO_2_ does not interfere with mechanical properties. Concerning the functionalization of the 3D printed structure, morphological analyses confirmed the presence of the photocatalyst on its surface, enhancing its potential for photocatalytic applications. By utilizing the structure with 1% TiO_2_ for testing photocatalytic activity, the effectiveness in degrading Rhodamine B (Rh_B_), defined as a model pollutant, was tested under controlled experimental conditions. The photocatalytic efficiency of the materials was evidenced by an observed 80% decrease in Rh_B_ concentration after 4 hours. Furthermore, the economic analysis indicates that 3D printing is the most cost-effective method for constructing reactors when the production quantity is relatively low, around 7000 units. In summary, this work illustrates a straightforward, cost-effective, and rapid method for immobilizing photocatalysts. Therefore, this approach allows for a more focused assessment of the photocatalytic performance of the printed material and provides valuable insights into its potential application in photocatalysis-related fields, such as environmental remediation or water purification.

## Data availability

The data supporting this article have been included as part of the ESI.†

## Author contributions

Isabel S. O. Barbosa: investigation, validation, methodology, writing – original draft. Yaidelin A. Manrique: investigation, validation, methodology, writing – review & editing. Diana Paiva: investigation, validation, methodology. Joaquim L. Faria: supervision, conceptualization, writing – review & editing. Ricardo J. Santos: supervision, conceptualization, investigation, validation, writing – review & editing. Cláudia G. Silva: supervision, conceptualization, investigation, validation, writing – review & editing.

## Conflicts of interest

There are no conflicts to declare.

## Supplementary Material

RA-015-D4RA07121B-s001

## References

[cit1] Castedo A., Mendoza E., Angurell I., Llorca J. (2016). Catal. Today.

[cit2] Okafor O., Weilhard A., Fernandes J. A., Karjalainen E., Goodridge R., Sans V. (2017). React. Chem. Eng..

[cit3] Qiu J., Gao Q., Zhao H., Fu J., He Y. (2017). ACS Biomater. Sci. Eng..

[cit4] Menzel V. C., Tudela I. (2022). Curr. Opin. Chem. Eng..

[cit5] Hernández-Afonso L., Fernández-González R., Esparza P., Borges M. E., Díaz González S., Canales-Vázquez J., Ruiz-Morales J. C. (2017). J. Chem..

[cit6] Kodama H. (1981). Rev. Sci. Instrum..

[cit7] Zhu J., Wu P., Chao Y., Yu J., Zhu W., Liu Z., Xu C. (2022). Chem. Eng. J..

[cit8] Wang H., Jiang G., Han Q., Cheng Y. (2021). Chem. Eng. J..

[cit9] Martin J. H., Yahata B. D., Hundley J. M., Mayer J. A., Schaedler T. A., Pollock T. M. (2017). Nature.

[cit10] Zhang Y., Zhang F., Yan Z., Ma Q., Li X., Huang Y., Rogers J. A. (2017). Nat. Rev. Mater..

[cit11] Bhattacharjee N., Urrios A., Kang S., Folch A. (2016). Lab Chip.

[cit12] Zheng M., Guo Q., Yin X., Getangama N. N., de Bruyn J. R., Xiao J., Bai Y., Liu M., Yang J. (2021). J. Mater. Chem. A.

[cit13] Waheed S., Cabot J. M., Macdonald N. P., Lewis T., Guijt R. M., Paull B., Breadmore M. C. (2016). Lab Chip.

[cit14] MacDonald E., Wicker R. (2016). Science.

[cit15] Parra-Cabrera C., Achille C., Kuhn S., Ameloot R. (2018). Chem. Soc. Rev..

[cit16] Capel A. J., Edmondson S., Christie S. D., Goodridge R. D., Bibb R. J., Thurstans M. (2013). Lab Chip.

[cit17] Konarova M., Aslam W., Ge L., Ma Q., Tang F., Rudolph V., Beltramini J. N. (2017). ChemCatChem.

[cit18] Martín de Vidales M. J., Nieto-Márquez A., Morcuende D., Atanes E., Blaya F., Soriano E., Fernández-Martínez F. (2019). Catal. Today.

[cit19] Bergamonti L., Bergonzi C., Graiff C., Lottici P. P., Bettini R., Elviri L. (2019). Water Res..

[cit20] Van Gerven T., Stankiewicz A. (2009). Ind. Eng. Chem. Res..

[cit21] Sagandira C. R., Siyawamwaya M., Watts P. (2020). Arab. J. Chem..

[cit22] Baumann M., Moody T. S., Smyth M., Wharry S. (2020). Org. Process Res. Dev..

[cit23] Bloemendal V. R. L. J., Janssen M. A. C. H., van Hest J. C. M., Rutjes F. P. J. T. (2020). React. Chem. Eng..

[cit24] Torres Arango M. A., Kwakye-Ackah D., Agarwal S., Gupta R. K., Sierros K. A. (2017). ACS Sustain. Chem. Eng..

[cit25] Lee C.-Y., Taylor A. C., Beirne S., Wallace G. G. (2017). Adv. Energy Mater..

[cit26] Jo W., Yoon B. J., Lee H., Moon M.-W. (2017). 3D Print. Addit. Manuf..

[cit27] Chen L., Tang X., Xie P., Xu J., Chen Z., Cai Z., He P., Zhou H., Zhang D., Fan T. (2018). Chem. Mater..

[cit28] He P., Tang X., Chen L., Xie P., He L., Zhou H., Zhang D., Fan T. (2018). Adv. Funct. Mater..

[cit29] Son S., Jung P. H., Park J., Chae D., Huh D., Byun M., Ju S., Lee H. (2018). Nanoscale.

[cit30] Elkoro A., Soler L., Llorca J., Casanova I. (2019). Appl. Mater. Today.

[cit31] Sevastaki M., Suchea M. P., Kenanakis G. (2020). Nanomaterials.

[cit32] Hansen A., Renner M., Griesbeck A. G., Busgen T. (2020). Chem. Commun..

[cit33] Mai Z., Liu D., Chen Z., Lin D., Zheng W., Dong X., Gao Q., Zhou W. (2021). Polymers.

[cit34] Do H. H., Tran T. K. C., Ung T. D. T., Dao N. T., Nguyen D. D., Trinh T. H., Hoang T. D., Le T. L., Tran T. T. H. (2021). J. Water Proc. Eng..

[cit35] Halevi O., Tan J. M. R., Lee P. S., Magdassi S. (2017). Adv. Sustainable Syst..

[cit36] Zhang L., Shi X., Zhang Z., Kuchel R. P., Namivandi-Zangeneh R., Corrigan N., Jung K., Liang K., Boyer C. (2021). Angew Chem. Int. Ed. Engl..

[cit37] Vyatskikh A., Kudo A., Delalande S., Greer J. R. (2018). Mater. Today Commun..

[cit38] Huang W., Mei H., Chang P., Pan L., Cheng L., Zhang L. (2022). Addit. Manuf..

[cit39] Chen C.-H., Wang S.-C., Chen H.-W., Chou T.-Y., Chang C.-S. (2024). ACS ES&T Water.

[cit40] Fry C. , Mihalko A., Michael R. and Piovesan D., presented in part at the Volume 3: Biomedical and Biotechnology Engineering, 2018

[cit41] Martin-Montal J., Pernas-Sanchez J., Varas D. (2021). Polymers.

[cit42] Li N., Tong K., Yang L., Du X. (2022). Mater. Today Energy.

[cit43] Hu C., Chang L.-L., Chen W., Hsu W.-Y., Chien S.-C., Chen C.-H., Lin Y.-T., Hsu T.-J., Tung K.-L. (2024). Chem. Eng. J..

[cit44] Guo J., Zeng Y., Li P., Chen J. (2019). Ceram. Int..

[cit45] Li K., de Rancourt de Mimérand Y., Jin X., Yi J., Guo J. (2020). ACS Appl. Nano Mater..

[cit46] Aguirre-Cortés J. M., Moral-Rodríguez A. I., Bailón-García E., Davó-Quiñonero A., Pérez-Cadenas A. F., Carrasco-Marín F. (2023). Appl. Mater. Today.

[cit47] Zhakeyev A., Jones M. C., Thomson C. G., Tobin J. M., Wang H., Vilela F., Xuan J. (2021). Addit. Manuf..

[cit48] Gomes N. M. O., Fonte C. P., Sousa C. C. e., Mateus A. J., Bártolo P. J., Dias M. M., Lopes J. C. B., Santos R. J. (2016). Chem. Eng. Res. Des..

[cit49] Melocchi A., Loreti G., Del Curto M. D., Maroni A., Gazzaniga A., Zema L. (2015). J. Pharm. Sci..

[cit50] zewczak A. S., zeląg M. S. (2019). IOP Conf. Ser. Mater. Sci. Eng..

[cit51] Chen S., Guo L. (2014). Chem. Eng. Sci..

[cit52] Pepper M. E., Seshadri V., Burg T. C., Burg K. J., Groff R. E. (2012). Biofabrication.

[cit53] Rai V., Mukherjee C., Jain B. (2017). Indian J. Pure Appl. Phys..

