# Supplementary material for: Efficient photocatalytic reactors via 3D printing: SLA fabrication and TiO2 hybrid materials
Source: RSC Adv. 2025 Jan 23;15(4):2275–86. doi: 10.1039/d4ra07121b (PMC11755327; doi:10.1039/d4ra07121b)
Supplement: RA-015-D4RA07121B-s001 [file RA-015-D4RA07121B-s001.pdf]

Supplementary Information (SI) for RSC Advances.
This journal is © The Royal Society of Chemistry 2025

ARTICLE

Additive Manufacturing of Photocatalytic Reactors

Isabel S.O. Barbosa,[a,c] Yaidelin J.A. Manrique, [a,c] Diana Paiva, [b,c] Joaquim L. Faria, [a,c] Ricardo J. Santos, [a,c] and Cláudia G. Silva *[a,c]

Received 00th January 20xx,
Accepted 00th January 20xx

DOI: 10.1039/x0xx00000x

Supplementary Information.

A Physical and Chemical Properties of TiO2 Aeroxide® P25 (Evonix)

Table 1 shows the characteristic physical and chemical data.

Table 1 Physical and Chemical Properties of TiO_2 Aeroxide® P25 (Evonix).

**Specific surface area (BET)**	35-65 m^2/g
**pH value** in 4% dispersion	3.5-4.5
**Loss on drying (2h at 105 °C)**	≤1.5 %
**Tamped density**	100-180 g/l
**Titanium Dioxide** based on ignited material	≥99.50%
**Al_2O_3** based on ignited material	≤0.300%
**SiO_2** based on ignited material	≤0.200%
**Fe_2O_3** based on ignited material	≤0.010%
**HCl** based on ignited material	≤0.300%
**Sieve residue (by Mocker, 45µm)**	≤0.050%

Please do not adjust margins

B Conditions of Rheological Tests

In rotational tests, all the resins were tested and classified under the conditions set in Table G.2.

Table 2 Experimental Conditions used in CSR and CSS Tests.

Test	R1	R2
**Type of curve**	Viscosity curve (VC)	Flow curve (FC)
**Temperature (°C)**	20	20
**Shear Rate $\dot{\gamma}$ (s^{-1})**	1-100	-
**Shear Stress τ (Pa)**	-	1-100
**Increasing**	linear	linear
**Measurement Time**	100 points<br>5 s/point	100 points<br>5 s/point

Table 3 Step conditions of the Temperature-dependent viscosity (TC) test.

	Pre-shear	*Ramp Up*	*Ramp Down*
**Temperature (°C)**	20	20-40	40-20
**Shear Rate $\dot{\gamma}$ (s^{-1})**	100	100	100
**Increasing**	-	Linear	-
**Measurement Time**	1 point<br>3 min/point	100 points<br>5s/point	3 points<br>2 min/point

This journal is © The Royal Society of Chemistry 20xx

Please do not adjust margins
